# Temporal relationship between perceptual and physiological events triggered by nociceptive heat stimuli

**DOI:** 10.1038/s41598-019-39509-3

**Published:** 2019-03-01

**Authors:** J. M. Castellote, J. Valls-Solé

**Affiliations:** 1 National School of Occupational Medicine, Carlos III Institute of Health and CIBERNED, Madrid, Spain; 2 Department of Physical Medicine and Rehabilitation, School of Medicine, Complutense University of Madrid, Madrid, Spain; 3 EMG and Motor Control Section, Neurology Department, Hospital Clinic, University of Barcelona, Barcelona, Spain

## Abstract

A combined assessment tool for the perceptual-motor aspects of pain processing will be valuable to clinicians. Fifteen healthy subjects were exposed to contact-heat stimulation (Pathway, Medoc, Israel) to assess perception through a simple task (motor response or conscious appraisal of the time the stimulus was felt) or with a dual task (both responses). The outcome measure was the temporal relationship between contact heat evoked potentials (CHEPS), reaction time (RT) and conscious awareness (AW). There were different temporal profiles for CHEPs, RT and AW to changes in stimulus intensity, AW being the least affected. Performing the dual task led to a significantly more pronounced effect on RT than on AW, while CHEPS were not influenced by task performance. Our results support the dissociation between physiological, behavioral and cognitive events elicited by nociceptive stimuli. The time of conscious appraisal of stimulus occurrence is a complementary information to other responses such as evoked potentials or behavioral tasks. The combined assessment of physiological and behavioral aspects of pain processing may provide clinicians with information on the different paths followed by nociceptive afferent inputs in the central nervous system.

## Introduction

The recording and analysis of contact heat evoked potentials (CHEPs) is a well-known method to examine conduction in pain pathways^1–3^. The stimulus implies a rapid increase of temperature in a thermofoil thermode applied over the skin at the desired site. The fastest possible temperature increase rate is 70°/s, which entails for slightly less than 300 ms for the temperature to increase from 32 °C to 52 °C. Onset of nociceptor activation depends on the steepness of the heating ramp^4^. However, due to the relatively long duration of such a stimulus, it is difficult to determine the exact point at which the skin receptors that generate CHEPs have received enough energy for their activation and generation of the propagated action potentials towards the CNS. In opposition to the almost immediate increase in temperature induced by laser stimulation at receptor level^5^, the progressive increase in temperature of the contact-heat probe may induce activation of receptors in various fibers up to those that generate CHEPs. This apparent disadvantage of contact-heat stimuli may allow for experimental tests on stimulus perception.

In normal conditions, any suprathreshold stimulus, including the temperature stimulus leading to CHEPs, can be consciously detected. However, it is challenging to determine when such conscious perception takes place in relation to the time of stimulus onset^6^. Theoretically, afferent volleys of inputs sufficiently synchronized as to activate the brain structures can be produced at any point beyond certain temperature level. In fact, there are many situations in work or social and domestic life indicating that the relationship between stimulus intensity and conscious perception of sensory signals is not linear. Examples can be found in the difficulties to judge if we are able to hold barehanded a hot container long enough to leave it safely on top of a distant table or whether or not a certain amount of sun exposure will burn our skin when lying on the beach.

Physiological events, such as CHEPs may reflect the precise time at which the inputs reach the brain, but long-latency event-related evoked potentials are contributed in part by endogenous inputs^7^ and stimulus salience^8^, and their latency is not necessarily related to the time of conscious perception^6^. Behavioral responses to stimulus perception, in the form of a reaction time (RT), is yet another variable with uncertain time reference, since RT is influenced by attention, fear, preparation and many other modifiers^9–11^. A means of assessing the time at which subjects become conscious of perceiving the stimulus can be done with the Libet’s clock^12^. With this method, subjects can state, with the accuracy of a normal clock divisions, the time at which they were consciously aware of stimulus perception (AW).

We reasoned that there would be a temporal relationship between latency of CHEPs, RT, and conscious perception of stimulus occurrence (AW), as they reveal successive steps in the central processing of thermo-algesic stimuli. Our aim was to investigate whether the temporal relationship among these events was fixed, following only the logical rule that, within certain limits, stronger stimuli lead to earlier responses of higher magnitude^13–16^, or it could be altered according to stimulus characteristics or the tasks in which subjects were engaged. The first option would indicate a lineal stream in successive steps of processing sensory inputs, while the second option would indicate the engagement in the process of a flexible and adaptable circuitry to prioritize certain events according to context.

**Figure 1:**
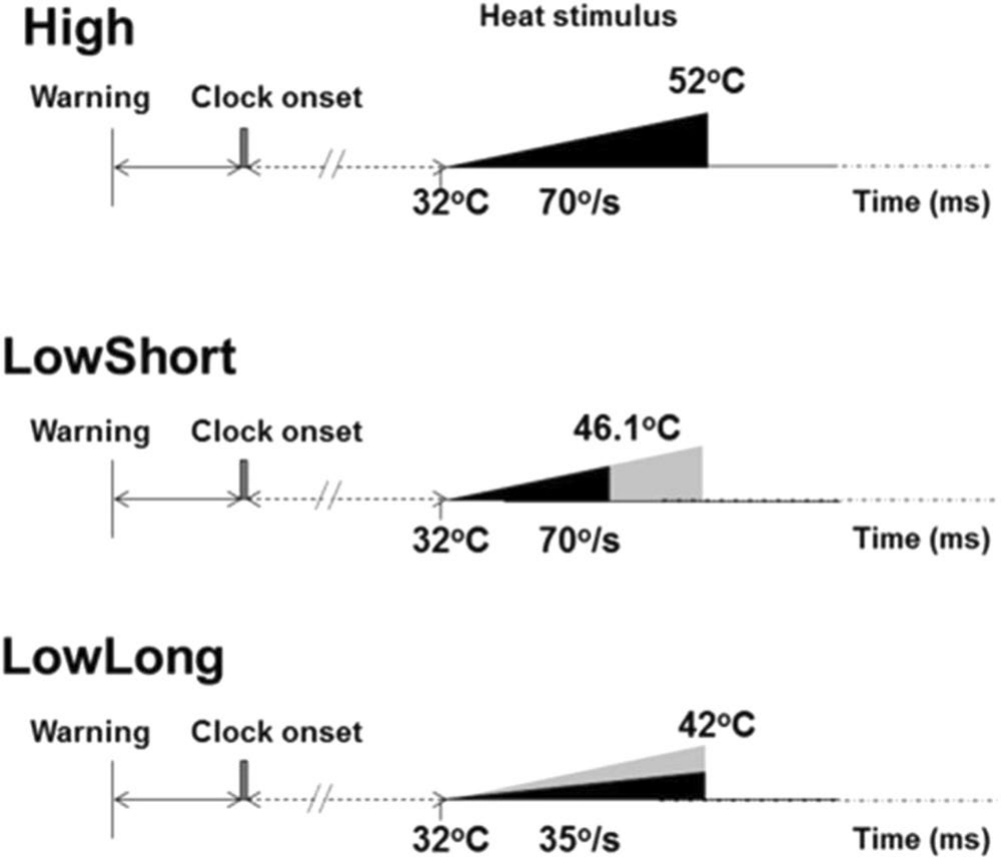
Experimental setup. After a warning signal, the Libet’s clock started running. At a random time after clock onset the stimulator delivered the heat stimulus at the predetermined intensity and duration. The area of black triangles represents the delivered thermal load (duration*temperature rise/2) for each condition. For representative purposes the end of the stimulus is represented as a vertical line, although the real stimulus ends with progressive reduction in temperature. The area of the black triangle representing thermal load in conditions LowShort and LowLong was half that of condition High, the grey shadow representing the missing part of the condition High. Condition LowShort had the same stimulus rate as condition High, while condition LowLong had the same time-to-peak as condition High.

We used different thermal stimulus loads modifying, within safety, temperature rise over time and duration. The thermal load was calculated finding the area underneath the triangular energy load profile (duration*temperature rise/2). The three stimulation conditions used in the study are defined as follows and depicted in Fig. 1:

- **High**. This was the stimulus with greatest intensity. The temperature rose from a basal value of 32 °C to a peak value of 52 °C at a stimulus rate of 70°/s. Duration of stimulus was 286 ms and, therefore, the thermal load was 2.86 °C*s. The parameters for the other two stimulation forms were calculated to carry a thermal load half the one used in this condition (i.e., 1.43 °C*s), keeping velocity of temperature increase in one case and duration in the other.
- **LowShort**. For a stimulus rate of 70°/s, we selected the peak temperature required to obtain a thermal load of 1.43 °C*s, which was 46.1 °C. Stimulus duration was, in this case, 201 ms.
- **LowLong**. For a stimulus duration of 286 ms, we selected the peak temperature required to obtain a thermal load of 1.43 °C*s, which was 42 °C. Velocity of temperature increase was, in this case, 35°/s.

The context in which the stimuli were given was also modified by asking the subjects to just assess AW (AW task), just react (RT task) or both responses (dual task, i.e., AW + RT).

## Results

All subjects performed the tests without difficulty and there were no adverse events. Only a few trials were repeated on-line because of subjects blinking, artifacts present in the EP recordings, or reduced attention self-reported by subjects (less than 1% of the total number of trials). The percentage of trials excluded from statistical analysis was similar for all sessions. A preliminary comparison between men and women gave no differences for AW, RT or CHEPs (p > 0.05 for all comparisons) and, therefore, the data shown correspond to data from both genders pooled together. The mean subjective score of pain using a visual analog scale from 1 to 10 (VAS) was 8.8 (SD = 0.8) in High trials and reduced to 5.4 (SD = 0.7) in LowShort and 4.9 (SD = 0.9) in LowLong trials.

### Characteristics of the CHEPs, RT and AW according to conditions and tasks

**Figure 2:**
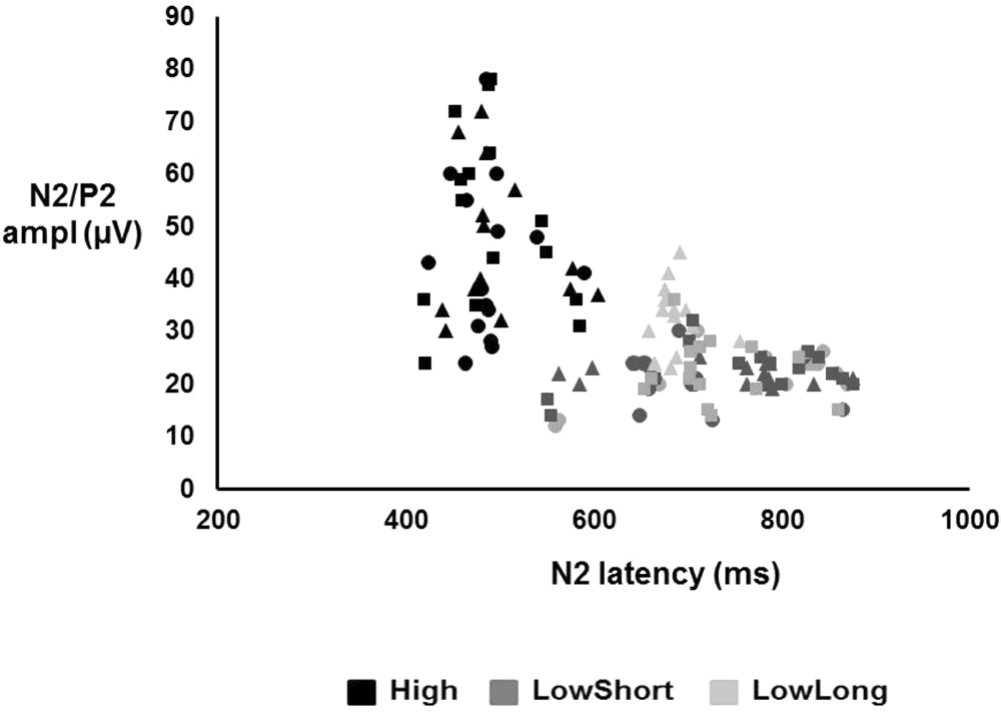
Graphical representation of CHEPs individual data for all conditions and tasks. Symbols represent mean values of individual CHEPs data (N2 latency vs N2/P2 amplitude). Symbols in black represent data in single task for AW, those in dark grey represent data in dual task and symbols in light grey represent data in single task for RT.

**Figure 3:**
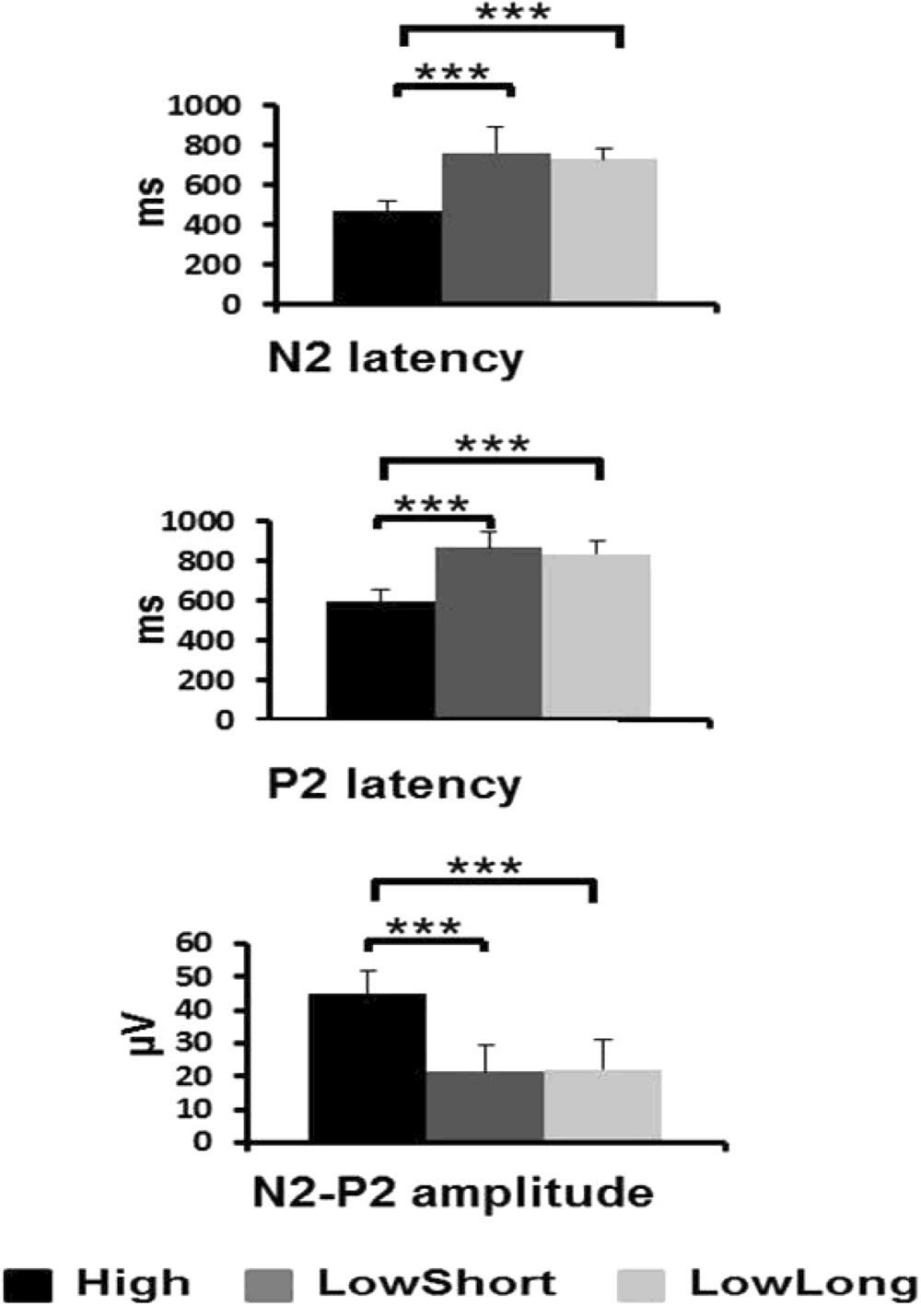
Group data (mean and SD) for N2 and P2 latencies, and N2-P2 amplitude, for each stimulation condition. Asterisks above the boxes define the level of significance for between-group comparisons by condition (***P < 0.001).

Mean and one SD values are presented in Table 1 for CHEPs, AW and RT. CHEPs were present in 92% of trials in the condition High, 77% in the condition LowShort and 80% in the condition LowLong. Figure 2 shows the individual N2/P2 peak amplitude values distributed along N2 latency values for all conditions. The N2 and P2 latencies were shorter and the N2/P2 amplitude was larger for the condition High than for the conditions LowShort and LowLong (Fig. 3). Two-factor ANOVA (task*condition) showed a condition effect for N2 latency (F_2,129_ = 132.02, *P* < 0.001), P2 latency (F_2,129_ = 178.41, *P* < 0.001) and N2-P2 amplitude (F_2,129_ = 52.49, *P* < 0.001), but not a task effect (p > 0.2 for all comparisons). Post-hoc analyses showed significant differences between High and both LowShort and LowLong conditions (*P* < 0.001 for both), but not between LowShort and LowLong (*P* > 0.2) for N2 and P2 latency and N2/P2 amplitude. There were no interaction effects between task and condition (p > 0.2 for all comparisons). An additional analysis done grouping those conditions with similar thermal load (LowShort and LowLong) and comparing them with High condition (two-factor ANOVA -condition*task-) showed a condition effect for N2 latency (F_1,56_ = 241.67, *P* < 0.001), P2 latency (F_1,56_ = 278.36, *P* < 0.001) and N2-P2 amplitude (F_1,56_ = 41.86, *P* < 0.001), with no significant task effect (p > 0.4 for all comparisons) or interaction (p > 0.3 for all comparisons).

**Table 1:** Data on evoked potentials and response variables for each condition and task.

	Condition	Single task	Dual task
N2 latency (ms)	High	478 (48)	463 (45)
	LowShort	775 (92)	758 (88)
	LowLong	741 (89)	721 (65)
P2 latency (ms)	High	599 (46)	585 (39)
	LowShort	879 (71)	859 (69)
	LowLong	846 (49)	826 (47)
N2-P2 amplitude (µV)	High	47 (10)	41 (8)
	LowShort	25 (9)	18 (8)
	LowLong	25 (7)	19 (9)
Reaction time (ms)	High	464 (65)	514 (91)
	LowShort	632 (72)	664 (88)
	LowLong	628 (57)	678 (82)
Awareness (ms)	High	342 (35)	358 (38)
	LowShort	393 (55)	424 (44)
	LowLong	384 (51)	421 (46)

Data are the mean and 1 SD value (in parenthesis) of evoked potentials, reaction time and awareness, for each condition and response task (single or dual). N2 refers to the largest negative peak, P2 refers to the largest positive peak and N2-P2 refers to peak-to-peak measurement.

**Figure 4:**
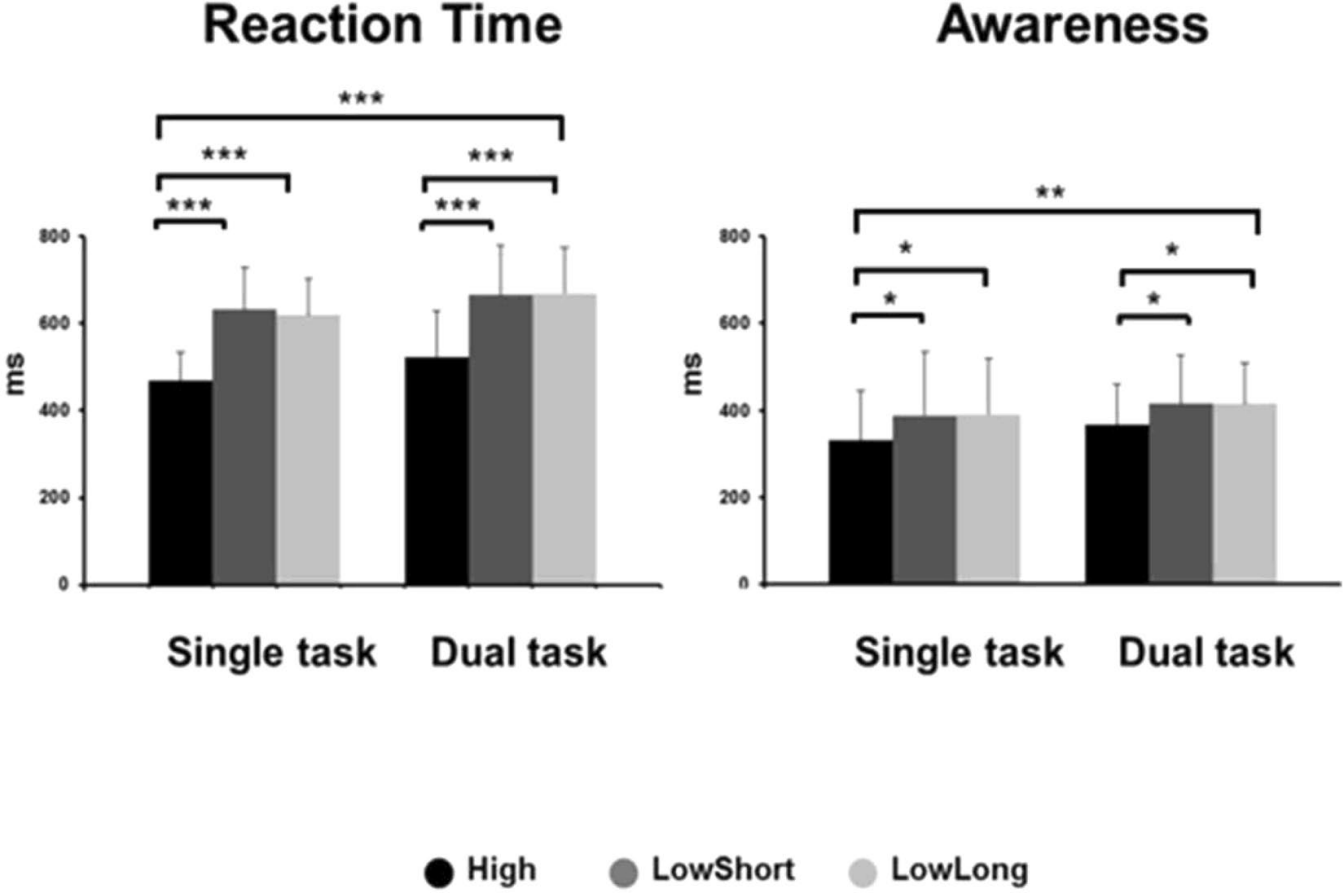
Group data (mean and SD) on reaction time and awareness for all conditions and tasks. The respective latencies of hand reaction time (**A**) and awareness (**B**) are represented for each type of task (single AW, single RT and dual), separated according to stimuli conditions (High, LowShort and LowLong). Note longer latencies for both, RT and AW when performing the dual task with respect to performing them as single tasks. Note that conditions LowShort and LowLong did not show much difference in spite of the different time resolution of the stimulus. Asterisks above the boxes define the level of significance of between-group comparisons ***P < 0.001).

RT was longer in conditions LowShort and LowLong than in condition High. It was also longer when subjects performed the dual task, i.e., reacting while assessing AW in comparison to when RT was the only task requested (Fig. 4A). Two-factor ANOVA (task*condition) showed a main condition effect (F_2,84_ = 26.45, *P* < 0.001), with shorter latencies for High condition with post-hoc differences between High and both LowShort and LowLong conditions (*P* < 0.001 for both), but not between LowShort and LowLong (*P* = 0.9). Subjects reacted faster in single task trials than in dual task trials (F_1,84_ = 12.12, *P* < 0.001). No condition*task interaction effects were present (F_2,84_ = 0.03, *P* = 0.9). The comparison of condition and tasks resulted in a condition effect for reaction time (F_1,56_ = 51.11, *P* < 0.001) and task (F_1,56_ = 11.12, *P* < 0.001), with no interaction (p > 0.8).

AW occurred always earlier than RT but, similarly, it was delayed in LowShort and LowLong conditions with respect to High condition and in dual with respect to single tasks (Fig. 4B). Two-factor ANOVA (task*condition) indicated a condition effect (F_2,84_ = 7.24, *P* < 0.01), with post-hoc differences showing shorter latencies for High than both LowShort and LowLong conditions (*P* < 0.05 for both), but not between LowShort and LowLong (*P* = 0.9). AW was of significantly shorter latency with single than with dual task trials (F_1,84_ = 7.45, *P* < 0.01). No condition*task interaction effects were present (F_2,84_ = 0.04, *P* = 0.9). The comparison of condition and tasks resulted in a condition effect on awareness (F_1,56_ = 13.14, *P* < 0.001) and task (F_1,56_ = 5.14, *P* < 0.01) with no interaction (p > 0.9).

### Temporal relationship between AW, EPs and RT

**Figure 5:**
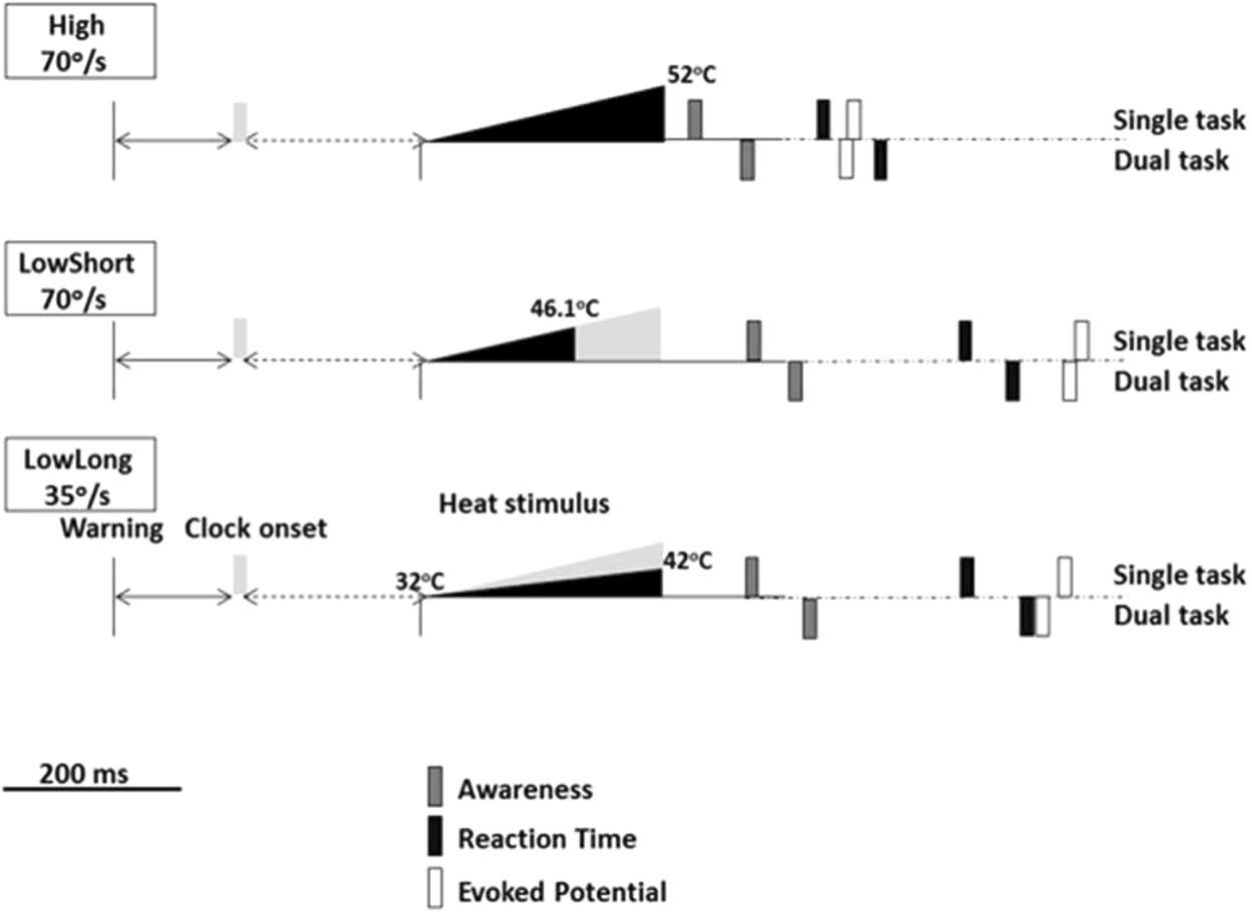
Changes in the timing of responses according to stimulation conditions. Approximate representation of the timing of events for the different stimulation conditions for single and dual tasks. Symbols as in Fig. 1.

**Figure 6:**
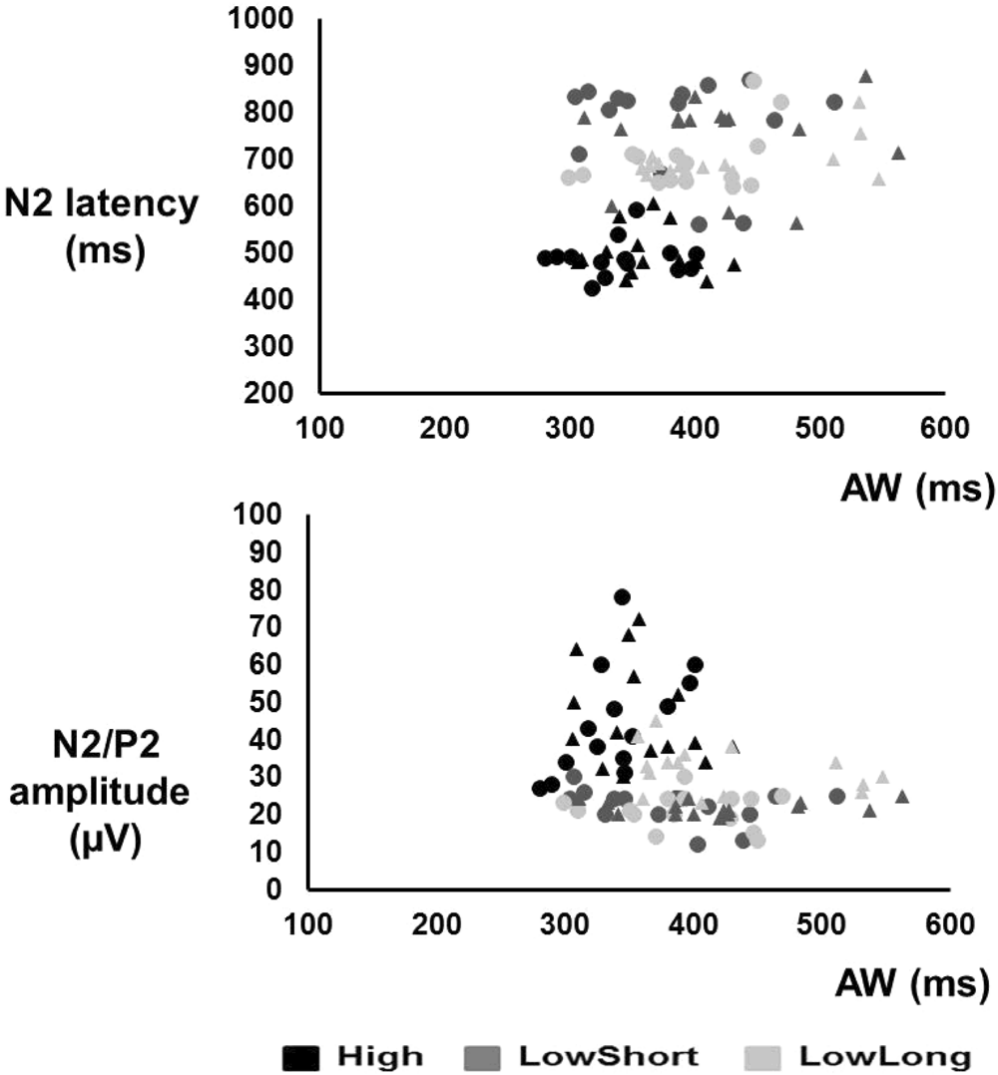
Time of AW plotted against N2 latency (**A**) and N2/P2 amplitude (**B**). Each symbol represents a subject for each stimulation condition for single task AW (circles) and dual tasks (triangles).

The three events occurred in different time delays with respect to stimulus onset. Usually AW was first, followed by RT and EP (Fig. 5). Pooling data from all conditions and tasks together, the differences in latency resulted statistically highly significant between AW and the other two variables (*P* < 0.001 for both comparisons) being just significant between EP and RT (*P* < 0.05). As expected, in all conditions and tasks, events occurred earlier with stimuli in High and single tasks than in the others. However, as previously analyzed, RT was highly dependent on condition and task, AW was just dependent on both, and EPs were highly dependent on condition but not dependent on task. We plot in Fig. 6 the distribution of individual AW values in relation to CHEPs (latency and amplitude) to illustrate the absence of a correlation.

### Latency cost of performing a dual task (AW + RT)

There was an increase in latency when performing the dual task in AW (59 ± 17 ms) and RT (76 ± 21 ms) (p < 0.05 for each, one sample *t*-test), and a residual no significant reduction in EP (19 ± 23 ms) (p > 0.05, one sample *t*-test). Performing both responses implied a significant difference in increases of latency among these variables (F_2,132_ = 109.31, *P* < 0.01). The post-hoc test revealed that the differences were between RT and AW (p < 0.05), RT and EP (p < 0.001), and AW and EP (p < 0.001).

## Discussion

The evoked potentials induced by contact heat stimuli (CHEPs) are very useful for the clinical study of pain pathways^1,6,17^. However, the contact heat stimulus has characteristics that differ from other stimuli used in neurophysiological study of evoked potentials. Its main characteristic is the progressive increase in temperature, which, in the most conventional stimulation protocols^3^ reaches the predetermined peak after some 300 ms. It is known that the time that receptors are exposed to a stimulus and the composition of the stimulus-induced afferent volley are important aspects for the processing of sensory inputs at the central nervous system^6,18,19^. In the study presented here, we have examined the effects of changes in the total time of exposure and energy of the stimulation on physiological, behavioral and cognitive responses. The main findings of the study are the different effects of stimulus intensity and task on the temporal profile of CHEPs, RT and AW.

The assessment of AW is not usually part of clinical neurophysiology testing. CHEPs are generated by dipoles representing the synchronization of EEG activity in various deep brain structures^20^. The size of CHEPs may be related to the degree of attention given to the stimulus^21,22^. However, CHEPs cannot be taken as a surrogate of conscious perception. Current evidence suggests that, in the nociceptive modality, the brain potentials evoked by a nociceptive stimulus are only partly related to the intensity of the perceived sensation^7,23^. RT is sometimes taken to indicate perception of the stimulus. A form of RT is used routinely in quantitative sensory testing for the determination of pain thresholds using the method of limits, in which the subject has to press a button when feeling the stimulus-related sensation^24,25^. However, conscious appraisal of the stimulus does not necessarily coincide with perception^6,26^. Subjects responding to external signals may rely on subcortical motor preparation^27^ and release the motor program, without full conscious perception of the sensation^9^. In some instances, behavioral responses follow stimuli that are neither overtly attended nor consciously detected^28–30^. Therefore, RT cannot be taken as a measure of conscious appraisal of the stimulus. In contrast, the Libet’s method brings the possibility to determine AW as the time the stimulus is felt after fixation in short term memory, which necessarily implies storage of the information in short-term memory domains and conscious retrieval of such information^6,31^.

Although CHEPs to low energy stimuli were difficult to record in a few trials, the three types of stimuli used in this study resulted in reproducible waveforms. As expected, stimuli with higher thermal load resulted in CHEPs of larger amplitude and shorter latency. While there is no doubt that the fibers conveying inputs to the central nervous system in our High condition were the Aδ fibers, the long latency of the N2 potential in LowShort and LowLong conditions might make the generator for those CHEPs more dubious. However, the VAS scores obtained in our subjects were within pain sensation levels, whereas inputs carried by C fibers are usually below pain threshold. This observation suggests that the Aδ fibers were those conveying the inputs to the CNS in our ‘Low’ conditions also. In fact, the available literature indicates that latencies of CHEPs may reach up to 800 ms, as after experimental nerve compression^32^ and the evoked potentials conveyed by C fiber to laser stimuli are usually beyond 800 ms^32,33^. Therefore, we think that the longer latency of CHEPs obtained in our study after activation of pain receptors with low energy loads is compatible with conduction velocities in the slower range of Aδ fibers, although we cannot completely rule out some contribution of C fibers. CHEPs, RT and AW changed according to the stimulation conditions, but the timing of each event differed in each condition. Each event shows different processing time, reflecting that they may travel in different processing streams. This shows the adaptability to context of circuits leading to the events recorded in this study. In the specific case of CHEPs, latencies and amplitudes depended mainly on stimulation conditions^6,30^. Attention is a key factor for determination of CHEPs and LEPs size^7,31,32,34^. In fact, in our study, attention was focused on responding, which might have been the reason why we have not found differences in the size of CHEPs among different study conditions. The fact that the subject had to voluntarily prepare either an estimation of the time of stimulus delivery, a fast motor response or both, did not modify CHEPs characteristics. Instead, there were significant differences in RT, suggesting that attention has been diverted at later relays with respect to perceptual processing.

RT is known to be directly related to the intensity of the imperative signal^6,13,14,35^. The level of perception required for RT is not the same as the one required for AW. In the present study the stimulus was unique, and, therefore, subjects had to divide attention when asked to perform a fast motor response and, at the same time, maintain in memory the timing of the stimulus. According to our results, subjects gave priority to stimulus judgement (AW) than to the motor reaction (RT).

There were important differences in the behavior of RT and AW to changes in stimulus characteristics. This indicates two different perceptual pathways related to attention. Although both, RT and AW, had longer latencies when the thermal load was low, the delay was much longer for RT than for AW, suggesting that subjects primed the route to conscious appraisal, shadowing the motor response. This observation supports the idea of different routes for reaction and appraisal of sensory inputs, according to the perception/action dual-stream processing model established by Milner and Goodale’s^36^ and the concept of prior entry, described by Titchener. In the model described by Milner and Goodale, conscious processing follows a ventral stream while no conscious processes follow a dorsal stream. In these circumstances, an attentional bias can occur, giving priority to one of the two entries. The perceptual traces accounting for the two responses might have followed different paths, prioritizing AW over RT. Conscious perception has usually been related with an inferotemporal cortex route (ventral stream). Instead, motor reaction has been related to the posterior parietal cortex (dorsal stream). Although the dorsal stream has been explored mainly for visual and auditory perception^37–40^, a similar organization has been proposed for the somatosensory pathways^41^. The posterior parietal cortex has been suggested as an important relay for detection of somatosensory stimuli^42^. Dorsal and ventral streams have also different roles in processing pain stimuli^43,44^. The dorsal stream would be related to spatial discrimination of the stimulus, while the ventral stream would be related to awareness of stimulus intensity. This supports the findings reported in previous studies that evaluated the processing of visual, auditory, and innocuous somatosensory information^39^. According to these results, we may consider that CHEPs and RT would have followed the dorsal route, while AW would have followed the ventral route.

The motor task we asked the subjects to prepare did not have the special conscious requirements that has the skilled grasping of an object. It did not either demand the subject to be especially conscious about the stimulus. It was an open loop action, close to pointing movements, similar to what is known as ‘automatic pilot’ for actions^35,39,45^. AW appeared earlier than RT or CHEPs. For the same perceptual stimulus this may be due to differences in central processing time. In fact, conscious perception is advanced with attended events^38^, which justifies the relatively short AW values found in our study.

The study has some limitations. CHEPS were recorded from a single cortical source and only the N2-P2 component of the nociceptive evoked potentials was analyzed. This is in fact the easiest component to record and probably the one that is related to relevant aspects of sensory processing of afferent inputs at cortical level. However, it is not the earliest component indicating the arrival of the volley to the brain. The N1, recorded over the parietal cortex would indeed be the first component in regard to cortical latency. In fact, though, the N1 is hard to record with CHEPs. Another limitation of our study in relation to CHEPs is the fact that we did not collect baseline data, i.e., CHEPs while subjects were not involved in any task. Theoretically, CHEPs amplitude and latency could be different at rest than during task performance^3,46^. However, our aim was to compare data obtained during execution of a task and, therefore, we planned to have all recordings during task performance. Nevertheless, our data fit with the expected latency and amplitude for CHEPs in persons of the same age and gender^3^. We have not found gender differences in any of the parameters measured. However, gender differences have been found in psychophysical testing^47,48^ and there could be gender differences in perception of thermo-algesic stimuli that we did not observed because of our relatively small sample. A larger study focused on such possibility would have been needed to investigate this aspect. Finally, AW was determined from subjective evaluation of the sensory inputs through the Libet’s clock. This is probably the best way to obtain numerical data out of a subjective sensation, but it only refers to the time domain and this is rather variable among conditions. We can only state that our data were consistent among the subjects and conditions used in this study. Temperature stimulation through Pathway is safe if used within the established safety limits of the operating machine (Medoc, Ramat Yishai, Israel). However, the reader should be warned of the possibility of skin burns^49^.

It can be concluded that this study has made a combined assessment of physiological, behavioral and cognitive responses in subjects involved in different tasks. The three events are inter-related but the relationship depends on many factors. Consciousness of stimulus perception is rather loosely correlated to behavioral and physiological responses and may be influenced by temporal binding between action and actor. The joint assessment of physiological, behavioral and cognitive responses may configure a kind of map of the sensory processing of a noxious stimulus, which can have clinical applicability in occupational medicine or in the study of patients with defective sensory or motor disturbances.

## Materials and Methods

### Subjects

Fifteen healthy subjects, 7 males and 8 females, aged 25 to 50 years (mean age 37.5 ± 7.4 years) took part in the study. They were recruited among staff members of our department. A preliminary interview was carried out to select only subjects that were free from any deficits that could affect the execution of the study and known neurological diseases affecting the sensory system. The experiment was performed with the understanding and written informed consent of each subject. The experimental procedures complied with relevant laws and institutional guidelines and were approved by the Ethical Committee of the Hospital Clinic of Barcelona.

### Set up

Subjects were sitting comfortably on an armchair in a dimly-illuminated room maintained at a stable temperature of 22 °C. Subjects were asked to keep their hands resting on top of a table. Heat stimuli were generated using Pathway (Medoc, Ramat Yishai, Israel). The thermode was gently applied to the ventral side of the distal part of the left forearm, and left in place for at least 10 seconds before delivering the actual stimulus^50^. We routinely changed the thermode position after each stimulus by placing it on an area that did not overlap with the one in which the previous stimulus was given, to avoid attenuation of heat perception. We applied the stimulus with a random interval that, for most trials, was between four and seven seconds counting from trial end -that is from AW verbal response or end of stimulation for trials without AW response- and application of the thermode over the skin. The stimulator consists of a fast heating/fast cooling probe of an area of 5 cm^2^, which rate of rise and peak temperature are computer-controlled.

We used a KeyPointNet electromyograph (Natus Medical Incorporated, U.S.A.) for recording of EEG and EMG activity. We set the apparatus to record epochs of 1 s from three sources with the appropriate setup for each type of activity recorded. The EEG activity was recorded from Cz, with reference to linked earlobes, to obtain CHEPs with a sensitivity of 20 µV/div and a bandpass filter between 0.1 and 50 Hz^3,34^. A ground electrode was placed around the forehead. Impedance was tested at least twice during the experiment and was always kept below 5 kOhm. The eyelid movements were recorded with an electrode located over the lateral canthus of the eye, referenced also to linked earlobes, to identify blinking artefacts with a sensitivity of 50 µV/div and a bandpass filter between 0.1 and 500 Hz. The EMG activity was recorded with surface electrodes attached over the right extensor carpi radialis muscle, a wrist extensor muscle (WE). Direct visual observation of the recordings in the electromyograph allowed us to repeat on-line those trials in which the CHEPs could have been interfered by spontaneous or reflex blinking. Apart from that, the electromyograph was connected to a computer for data storage and off-line analysis. We recorded 20 single sweeps of 2 s with a 500 ms pre-stimulus delay for each condition (see below).

To assess AW, we used the Libet’s clock^12^. Subjects were looking at a clock face presented on a computer screen situated at eye level at a distance of 1 m. The clock had conventional 5 minute markings and a single hand, 1.3 cm long, which rotated every 2560 ms. All trials began with a verbal warning, calling for attention. Then, the experimenter pressed a computer’s key to send a 5 V trigger pulse out to start the sweep of the EMG machine and deliver the temperature stimulus from the Pathway. Subjects were instructed to retain in memory the position of the clock hand at the time they first felt the thermal pain with an accuracy of the 60^th^ division, as in a normal clock. At the end of each trial the experimenter entered the number given by the subject as the clock’s hand position subjective judgment, while the computer generated automatically the actual clock’s hand position at the onset of stimulus delivery. Each division of the clock’s face corresponds to 42.67 ms, which would be the temporal resolution error for individual trials.

We divided the study in three experimental sessions, each of them with a different task and presented in random order across subjects, one day apart.

In the DETECT session subjects were asked to just report the position of the clock hand at perception of the stimulus-related sensation (AW task).

In the REACT session, the subjects had to just react, performing a wrist extension movement to the perception of the stimulus, with no request for AW (RT task).

In the DETECT-REACT session, subjects had to perform both tasks, i.e., wrist extension movement in the reaction time task context, and retain in memory the position of the clock’s hand at perception of the stimulus-related sensation (RT + AW task).

### Procedure and test sequence

Subjects were allowed to practice to get accustomed to the experiment. Each session contained three blocks of trials (20 trials/block), corresponding to one stimulation condition:

- **High:** A stimulus rate of 70°/s, peak temperature of 52 °C, thermal load of 2.86 °C*s and stimulus time to peak of 286 ms.
- **LowShort**, With a stimulus rate of 70°/s, peak temperature of 46.1 °C, a thermal load of 1.43 °C*s and a stimulus time to peak of 201 ms.
- **LowLong**, With a stimulus rate of 35 °C/s, peak temperature of 42 °C, thermal load of 1.43 °C*s and a stimulus duration of 286 ms.

Stimulus intensities were scored by subjects using a VAS from 1 to 10 in nine trials (three for each condition) that preceded the start of each session, then the subjects concentrated in the session trials just on the required AW and RT responses. They were instructed that a mark of 4 would mean a slight pain sensation and a mark of 10 would be an unbearable pain sensation^1^. Subsequent blocks of trials were separated by periods of at least 5 minutes. To avoid fatigue, the experimental sessions were separated with breaks of 30–60 minutes.

### Data processing and analysis

Variables of interest (CHEPs, RT and AW) were analysed off-line for each trial, and the values grouped according to stimulus condition (High, LowShort, LowLong) and response task (RT task, AW task, RT + AW task). We considered time 0 the onset of the temperature stimulus. For the EPs we divided the 20 trials in 2 series of 10 averaged traces that were superimposed for consistency. We calculated the latency of the negative and positive peaks (N2 and P2), as well as the peak-to-peak amplitude (N2-P2), in the largest peak out of the two averaged waveforms. We also calculated persistence as the number of trials in which the EP could be recognized in percentage of the total number of stimuli applied in that condition. For recognition of the CHEPs in individual traces, we admitted their presence when there was a waveform of at least 5 μV peak-to-peak amplitude, which peak latency coincided with the averaged waveforms. For RT, EMG onset latency was measured in the recordings from the WE at the time when EMG activity exceeded 2 standard deviations (SD) of a 200 ms pre-stimulus baseline. AW was determined as the time difference between the subjective appraisal of clock’s hand position at perception of the thermal pain and the actual clock’s hand position at delivery of the stimulus, transformed in ms. This value includes, obviously, the time taken for the stimulus to activate the skin receptors as well as the peripheral conduction time, which were also included in the determination of RT and latency of CHEPs.

For descriptive purposes, raw values were used for each variable. For statistical comparisons normalized values were used. For EP, we normalized the data for each subject by assigning 100% to the mean value calculated for responses obtained in the condition High at the RT + AW task trials. RT was normalized to the mean value for RT in the condition High at RT task trials, and AW was normalized to the values obtained for AW in the condition High at AW task trials. We considered that the double RT + AW task could imply a cost in the form of time delay for RT and AW responses with respect to when the tasks were carried out separately. Therefore, we determined such time cost for either AW or RT by subtracting, for each individual, respectively, AW in the AW task from AW in RT + AW task, and RT in RT task from RT in RT + AW task.

Statistical comparison was carried out using a two factor ANOVA, one factor being stimulus condition (three levels, High, LowShort and LowLong) and the other being task (two levels, single task and dual task, i.e., AW + RT). Post-hoc comparisons between specific trial types were performed using the Scheffé test. The level of significance was set at p = 0.05.

## Data Availability

All relevant data are within the paper.
